# Using Global Analysis to Extend the Accuracy and Precision of Binding Measurements with T cell Receptors and Their Peptide/MHC Ligands

**DOI:** 10.3389/fmolb.2017.00002

**Published:** 2017-01-31

**Authors:** Sydney J. Blevins, Brian M. Baker

**Affiliations:** Department of Chemistry and Biochemistry and the Harper Cancer Research Institute, University of Notre DameNotre Dame, IN, USA

**Keywords:** T cell receptor, peptide/MHC, binding, mutagenesis, global analysis, double mutant cycle

## Abstract

In cellular immunity, clonally distributed T cell receptors (TCRs) engage complexes of peptides bound to major histocompatibility complex proteins (pMHCs). In the interactions of TCRs with pMHCs, regions of restricted and variable diversity align in a structurally complex fashion. Many studies have used mutagenesis to attempt to understand the “roles” played by various interface components in determining TCR recognition properties such as specificity and cross-reactivity. However, these measurements are often complicated or even compromised by the weak affinities TCRs maintain toward pMHC. Here, we demonstrate how global analysis of multiple datasets can be used to significantly extend the accuracy and precision of such TCR binding experiments. Application of this approach should positively impact efforts to understand TCR recognition and facilitate the creation of mutational databases to help engineer TCRs with tuned molecular recognition properties. We also show how global analysis can be used to analyze double mutant cycles in TCR-pMHC interfaces, which can lead to new insights into immune recognition.

## Introduction

T cell receptors (TCRs) are clonotypic membrane proteins on the surface of T cells. TCRs are responsible for recognizing peptide antigens bound and “presented” by major histocompatibility complex (MHC) proteins. TCR recognition of a peptide/MHC complex (pMHC) drives the initiation and propagation of a cellular immune response as well as the development and maintenance of the T cell repertoire. TCR recognition of pMHC is also involved in conditions such as autoimmunity and transplant rejection and is central to new biologic and cellular therapies for cancer and infectious disease. Given the central role these interactions play in human health, there has been significant interest in the physical mechanisms underlying TCR-pMHC recognition.

**Figure 1:**
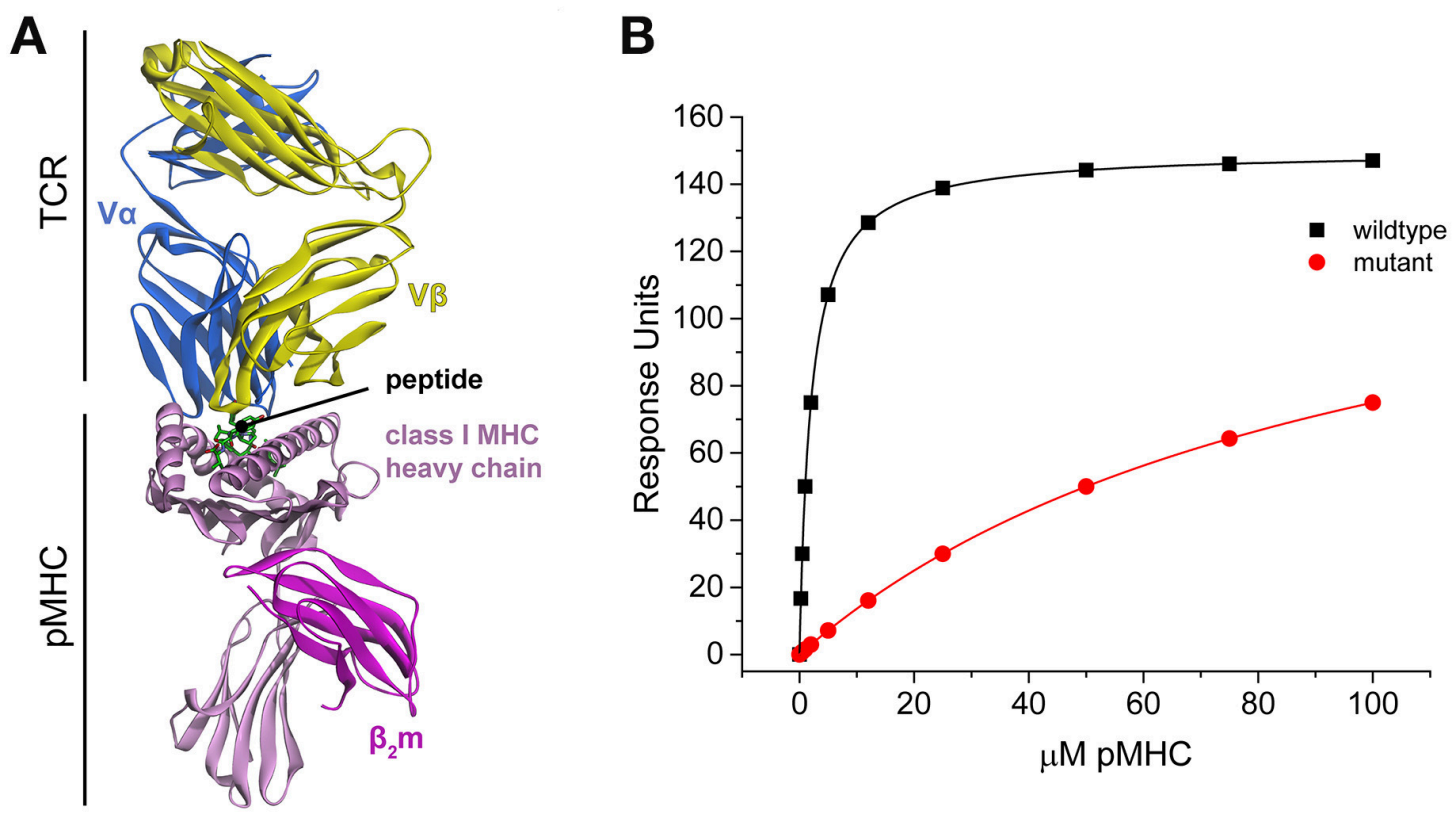
**TCR-pMHC structural overview and simulated binding data. (A)** Structure of a TCR-pMHC complex, showing the TCR bound to the composite pMHC ligand. **(B)** Simulated TCR-pMHC binding data. The wild-type data is simulated with a *K*_D_ of 2 μM and reaches 99% saturation, and mutant data with a *K*_D_ of 100 μM reaches ~50% saturation.

The TCR–pMHC interaction is marked by the structural and genetic complexity of its interface (Miles et al., 2015). Structurally, TCRs are similar to antibodies, comprising four immunoglobulin domains and an antigen binding site with complementarity determining region (CDR) loops generated through genetic recombination and nucleotide editing. However, TCRs and antibodies differ in the nature of the antigen they recognize. Whereas antibodies can be elicited against antigens of nearly unlimited structural diversity, TCRs recognize a composite surface consisting of the antigenic peptide bound in a groove formed of flanking α helices and a β sheet floor (Figure 1A). The peptide contributes ~30% of the recognized surface (Rudolph et al., 2006), indicating a significant contribution from the MHC to binding. This combined recognition of a highly diverse non-self-component (the peptide) in the context of a less-diverse self-component (the MHC) is a fundamental aspect of cellular immunity (Zinkernagel and Doherty, 1974).

To engage this combined ligand, the TCR often, although not exclusively, aligns its most diverse “hypervariable” CDR3 loops over the peptide, and the less diverse “germline” CDR1/CDR2 loops over the α helices of the MHC peptide binding groove. This alignment, however, does not always translate into interatomic contacts, as numerous contacts are typically formed to the peptide by germline loops and contacts to the MHC by hypervariable loops. Understanding the “roles” played by the different loops, the peptide, and the MHC protein in driving TCR selection of ligand and ultimately T cell biology has remained a long-standing immunological challenge, and has become even more relevant with the advent of engineered TCRs as therapeutic reagents.

Much of the current understanding of TCR recognition of pMHC has come from the growing collection of structures and subsequent interrogation by binding experiments (Rossjohn et al., 2015). Mutational analysis is regularly used to probe the contributions of different components of the TCR and pMHC. Alanine scanning mutagenesis has revealed binding “hot spots” on TCR and MHC surfaces (e.g., Manning et al., 1998; Baker et al., 2001; Borg et al., 2005). More targeted mutagenesis has been used to explore particular hypotheses (e.g., Wu et al., 2002; Feng et al., 2007; Borbulevych et al., 2009; Piepenbrink et al., 2009a), or in conjunction with computational design, generate variant TCRs with engineered binding properties (Zoete et al., 2013; Malecek et al., 2014; Pierce et al., 2014; Riley et al., 2016). Recently, double mutant cycles have been used to explore TCR recognition of pMHC (Piepenbrink et al., 2013; Blevins et al., 2016). Common to these and dozens of other mutational studies is quantitative assessment of the impact mutations have on binding energy, or the ΔΔG°.

A challenge in studying TCR-pMHC binding is that the affinities are generally weak, with *K*_D_-values in the micromolar range or weaker (Matsui et al., 1994; Davis et al., 1998; Cole et al., 2007). Weak affinities can lead to difficulties in measuring the effects of mutations, resulting in inaccurate and imprecise ΔΔG°-values. Although “thresholds” can be helpful (i.e., the ΔΔG° is greater than a cutoff; Borg et al., 2005), accurate measurements permit better comparison with structural information and relationships between structural properties and binding energies. With inaccurate measurements, incorrect conclusions about the functional roles positions play in TCR binding and T cell biology can even be made (Gagnon et al., 2005).

Fortunately, there are ways to design binding experiments that avoid the common pitfalls of low affinity interactions. A commonly applied method is to perform a global analysis of multiple data sets collected simultaneously (Beechem, 1992). This can be used with a range of experimental approaches, such as titration calorimetry and surface plasmon resonance (SPR), two common methods for studying TCR–pMHC interactions. Another approach is to fix an unknown experimental parameter by pre-determining it in a separate experiment. For example, in SPR, pre-determining the functional density of a sensor chip and constraining this parameter in subsequent experiments allows measurements with fixed surface densities that reach only 30% saturation to have the accuracy of those that reach 90% saturation when both ΔG° and surface density are fitted parameters (Piepenbrink et al., 2009b). Combined, global analysis of multiple datasets with constrained (or shared) experimental parameters should be expected to allow further gains in accuracy. Thus, appropriate experimental design and analysis can be used to obtain accurate ΔΔG°-values for very low affinity interactions, such as those seen with mutant TCRs and pMHC complexes.

Here, we used simulated and real TCR binding data to show how accurate and precise ΔΔG°-values can be obtained for very low affinity TCR–pMHC interactions. We demonstrate that global analysis with shared parameters significantly increases accuracy and precision, and can easily be applied to low affinity interactions. We also show how this procedure can be extended in the design and execution of double mutant cycle experiments, which can be used to probe TCR-pMHC interfaces and determine the contributions of various regions in more detail than traditional single amino acid mutagenesis (Piepenbrink et al., 2013; Blevins et al., 2016).

## Results and discussion

### Global analyses yield more accurate and precise ΔΔG° measurements for perturbed data

Global analysis of multiple datasets with shared parameters has long been used to extend the accuracy and precision of binding experiments (Beechem, 1992). To establish a baseline of the applicability of this approach for studying TCR–pMHC interactions, we simulated TCR-pMHC binding data reflective of a TCR-pMHC binding experiment that uses SPR to determine the ΔΔG° associated with a MHC or peptide mutation (Figure 1B). The *K*_D_ for the wild-type measurement was set at 2 μM (ΔG° = −7.77 kcal/mol), characteristic of a “high affinity” TCR recognizing a viral antigen. The *K*_D_ for the mutant measurement was set at 100 μM (ΔG° = −5.45 kcal/mol). The TCR surface activity (RU_max_) was set at 150 response units (RU). Data for both datasets included 10 pMHC injections over the concentration range of 0.25–100 μM. The wild-type dataset reached >99% saturation, whereas the mutant dataset reached only ~50% saturation.

The simulated data was then fit individually to determine the ΔG° for the wild-type and mutant data and thus the ΔΔG°. Individual fits floated both RU_max_ and ΔG°. The same two datasets were also fit globally, in which the RU_max_ was a global (or shared) parameter and the two ΔG°-values were local parameters. The ΔΔG°-values determined either way were indistinguishable, and identical to the actual value of 2.32 kcal/mol, with negligible error. This is expected as the simulated data were unperturbed, lacking the experimental noise that would arise in real experiments. To better test the robustness of global vs. individual analysis, a series of perturbations were added to the data to simulate noise/error arising from the instrument or user. To ensure that any introduced noise would be reflective of that seen in real experiments, the reduced χ^2^-values from 50 separate, individually fit actual TCR-pMHC SPR binding experiments (described below) were averaged, yielding a value of 22%. Based on this value, Gaussian-distributed random noise between 5 and 30% of the value of each data point was applied to the simulated data in a series of steps. This yielded 10 sets of perturbed wild-type and mutant datasets with increasing noise as shown in Table 1.

**Table 1:** **Data perturbations to simulate noise and error**.

**Perturbation name**	**Effect on data**
Normal	No change
5^a^	5% Gaussian distributed noise
7	7% Gaussian distributed noise
10^a^	10% Gaussian distributed noise
12	12% Gaussian distributed noise
15^a^	15% Gaussian distributed noise
18	18% Gaussian distributed noise
20	20% Gaussian distributed noise
22^b^	22% Gaussian distributed noise
25	25% Gaussian distributed noise
30	30% Gaussian distributed noise
W1	First three wild-type points removed
W2	Middle three wild-type points removed
W3	Last three wild-type points removed
M1	First three mutant points removed
M2	Middle three mutant points removed
M3	Last three mutant points removed

a *Used on experimental data sets*.

b *Average seen in experimental data*.

**Figure 2:**
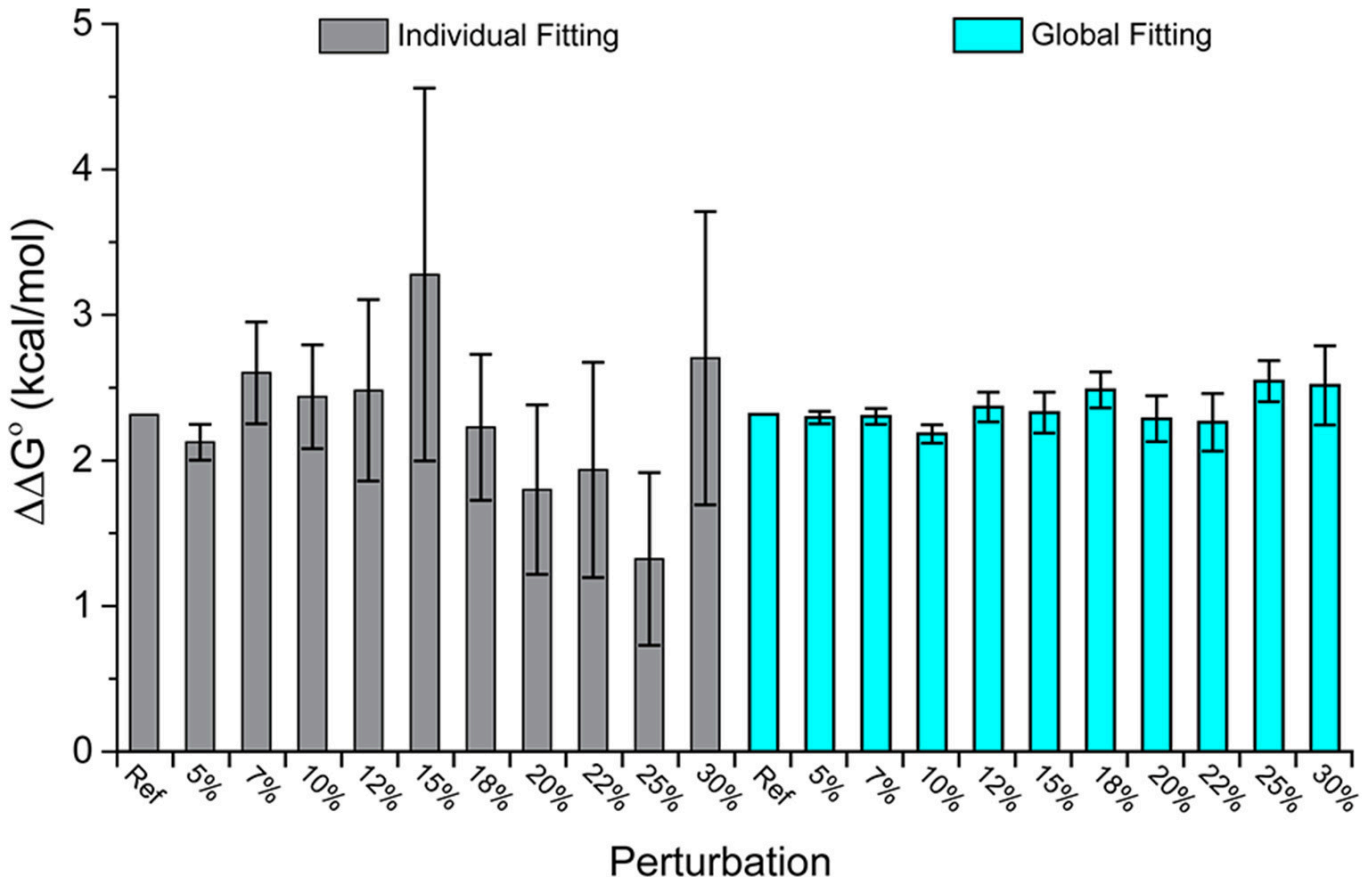
**Global analysis of simulated wild-type and mutant datasets perturbed with added noise outperforms single analyses**. While the average value of both is close to the real value, the average error of the global fits is 0.11 kcal/mol, compared to 0.51 kcal/mol for the individual fits. The columns correspond to increasing amounts of added noise as indicated; “Ref” refers to the actual ΔΔG° of 2.32 kcal/mol.

Figure 2 shows the ΔΔG°-values obtained from individual and global fitting of the pairs of perturbed wild-type and mutant simulated datasets. While the average ΔΔG°-value for both individual and global fitting sets is similar (individual fitting average of 2.29 kcal/mol, global fitting average of 2.35 kcal/mol), global fits more faithfully reproduced the actual value of 2.32 kcal/mol and showed far less variance across the 10 datasets (standard deviation of 0.51 kcal/mol from individual fitting, almost 5-fold larger than the standard deviation of 0.11 kcal/mol from global fitting). The error associated with global fitting was substantially smaller than the error associated with individual fitting (average of 0.56 kcal/mol for individual fitting, average of 0.18 kcal/mol for global fitting). *F*-tests confirmed that the variances of the values and their error differed significantly. Thus, with appropriate experimental design, global fitting is both more accurate and precise for determining ΔΔG°-values.

### Discrepancies between individual and global fits for experimental determinations of ΔΔG°-values

We next examined individual vs. global fitting for determining ΔΔG°-values in actual experimental data. We used the two fitting approaches with data for 25 pairs of wild-type/mutant binding experiments from three different TCR-pMHC interfaces. The data, comprising 50 separate titrations, were from SPR experiments in which wild-type or mutant pMHC was injected over the same TCR surface, facilitating parameter sharing for surface activity. The complexes analyzed included both published and unpublished data for the A6, DMF5, and Mel5 TCRs interacting with HLA-A*0201 presenting the Tax (for A6) or MART-1 (for DMF5 and Mel5) peptides, with various mutations or peptide substitutions (Table S1 and Figure S1; Piepenbrink et al., 2013; Blevins et al., 2016). In some cases, TCR mutations or peptide variants were used that enhanced affinities (Piepenbrink et al., 2009a; Ekeruche-Makinde et al., 2012; Cole et al., 2014; Pierce et al., 2014).

**Figure 3:**
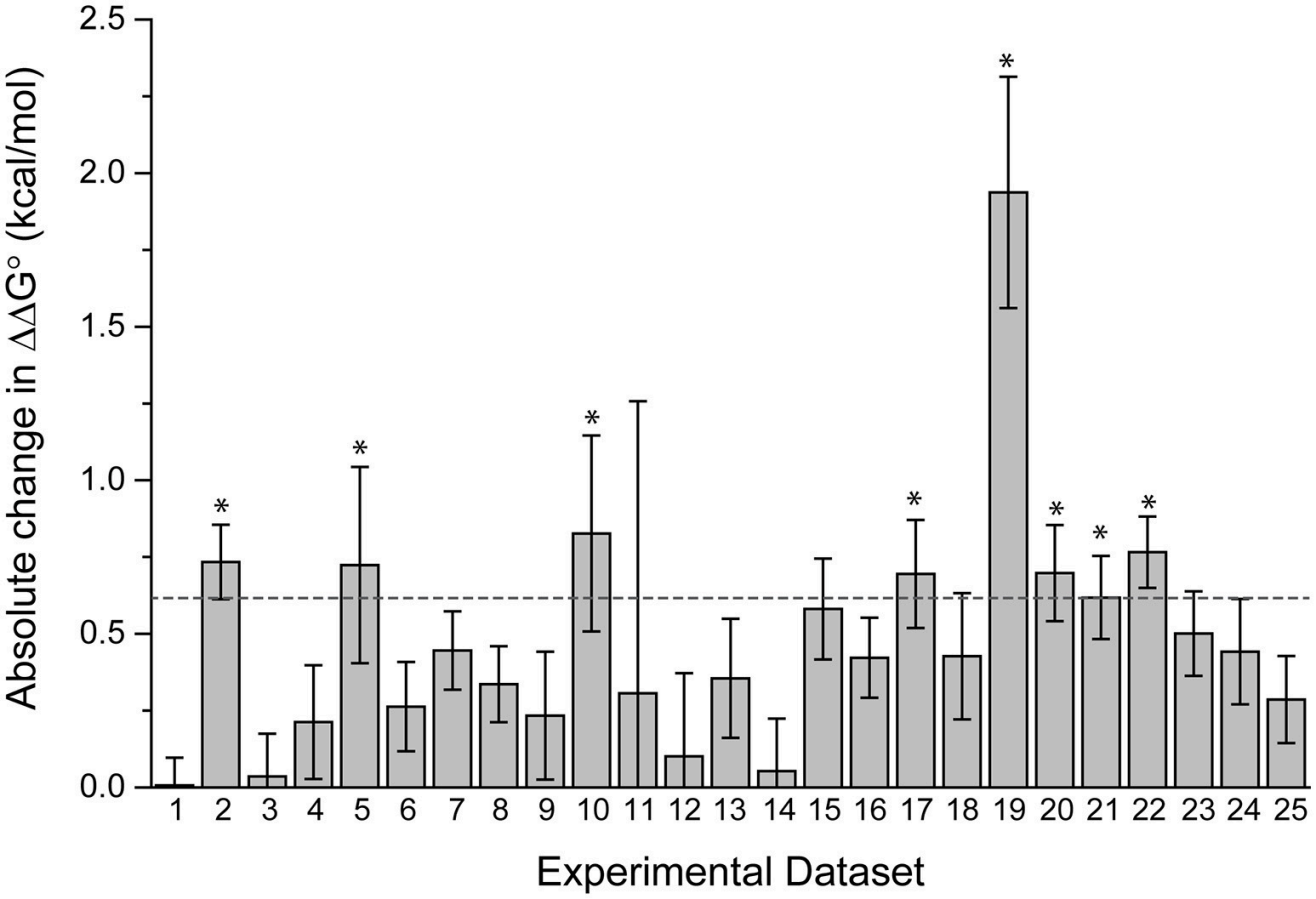
**Fitting wild-type and mutant experimental data individually or globally can lead to significant differences in the ΔΔG°**. The absolute value of the difference between individual and global fits, referred to as in the text as |Δ(ΔΔG°)| is shown for 25 pairs of experimental datasets. The dashed line represents a Z-score of 1.64 (90% cutoff of one standard deviation), revealing the eight datasets where the difference between individual and global fitting was most significant (noted by asterisks).

The datasets spanned a wide range of affinities, with individual fits giving *K*_*D*_-values ranging from nanomolar to micromolar. For the 25 pairs of data, we determined the ΔΔG°-values using both individual and global fits as above, and calculated the absolute value of ΔΔG° global fit – ΔΔG° individual fit, referred to as |Δ(ΔΔG°)|. As shown in Figure 3, there is wide variation in |Δ(ΔΔG°)|. Some datasets showed essentially no difference between individual and global fitting (e.g., datasets 1, 3, 14), while others had much more substantial differences (e.g., datasets 10, 19). The average |Δ(ΔΔG°)|-value among the 25 pairs of experiments was 0.48 kcal/mol. To determine cases of particular interest, a 90% confidence cutoff of the standard deviation, equal to a Z-score of 1.64, was used. Eight datasets fell outside of this range, which amounts to a |Δ(ΔΔG°)| of 0.62 kcal/mol (asterisks in Figure 3).

There was no correlation between the eight datasets with |Δ(ΔΔG°)| ≥ 0.62 kcal/mol and the apparent affinities of one or more of the binding experiments, as these sets included apparent affinities ranging from 100 nM to 200 μM. The range of the measured ΔΔG°-values was similarly wide, ranging from small (0.05 kcal/mol) to large (3 kcal/mol). The remaining 17 datasets were also dispersed within these ranges. This suggests that the large differences in fitting methods for the nine outlier datasets result from the influence of noise and error that is unique to individual experiments, as opposed to being directly correlated with strength of binding or ΔΔG°.

### Effects of additional noise and missing data points on experimental data

To further probe the differences seen between individual and global fitting, the eight experimental datasets with large ΔΔG° discrepancies when fit with individual vs. global fitting were further perturbed. Additional Gaussian error was introduced in stepwise increments (5, 10, and 15%) to both wild-type and mutant datasets as described above, and partial datasets for either wild-type or mutant were generated by deleting three consecutive points at the beginning, middle, and end of the wild-type or mutant titrations (Table 1; missing points may arise, for example, when bubbles occur during a sample injection). For all eight experimental datasets, this generated nine additional pairs (three pairs with noise added to wild-type and mutant data, three pairs with deleted data in the wild-type experiment, and three pairs with deleted data in the mutant experiment). The resulting 72 highly perturbed datasets were fit individually or globally to determine ΔΔG°-values.

For all eight datasets, the ΔΔG°-values for individual fitting were once again highly sensitive to perturbations, whereas the global fits were more robust. The individually fit ΔΔG°-values differed from their original, non-perturbed values by anywhere from 51 to 123%. For global fitting the range was 0.1–38%. The standard deviation of the average ΔΔG°-values for the individual fits was 0.65 kcal/mol, 5-fold greater than the value of 0.14 kcal/mol for global fits.

**Figure 4:**
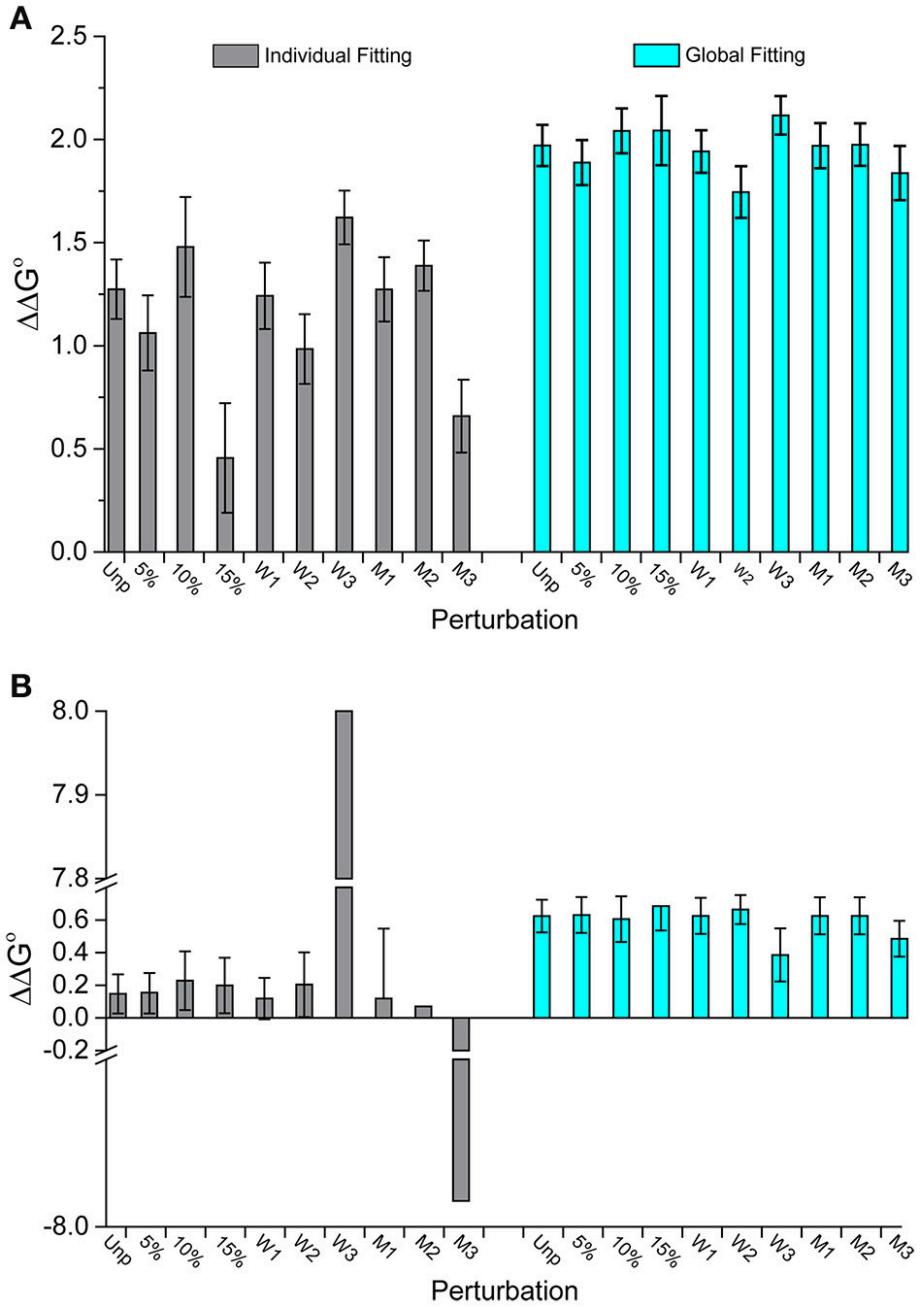
**Perturbations to experimental datasets can significantly impact accuracy and precision whereas global analysis is substantially more robust. (A)** The impact of added noise and deletion of data points for dataset 17 in Figure 3. Unlike individual fitting, global fitting handles the perturbations robustly. **(B)** A similar result is seen for dataset 2 in Figure 3. Of note are the instances in which perturbations to the last three wild type data points were removed (W3) or the middle three mutants removed (M3), in which the ΔΔG°-values were dramatically impacted for single fitting. The same perturbations however had little impact with global fitting.

Figure 4 illustrates this outcome for two datasets. In Figure 4A (dataset 17), the individually fit data shows much larger deviations from the original ΔΔG°-value than the globally fit data. The standard deviation of the ΔΔG°-values is three times larger for the individually fits compared to the global fits (0.3 kcal/mol for individual vs. 0.1 kcal/mol for global). Figure 4B (dataset 2) shows another example of discrepancies in ΔΔG°-values from the two fitting methods. Two of the perturbed series fit individually have excessively large ΔΔG°-values (+8 and −7.9 kcal/mol). These are undoubtedly incorrect and highlight the propensity of individual fitting to yield gross inaccuracies with noisy data. In comparison, all nine of the global fitting perturbation series showed reasonable ΔΔG°-values with a standard deviation of only 0.08 kcal/mol. Notably, the two datasets that were wildly inaccurate when fit individually were not distinctive when fit globally.

### Inaccurate single fitting stems from overfitting of lower affinity measurements

It is notable that the average ΔΔG° for all 25 datasets was smaller for individual fitting than global fitting (0.81 kcal/mol for individual vs. 1.25 kcal/mol for global). This was also reflected in the average ΔΔG°-values for the eight “outlier” datasets in Figure 3. Upon examining the results of each trial in the series of perturbed experimental datasets, it became clear that a significant portion of the differences between individual and global fitting could be attributed to fitting of the lower-affinity mutant data. In most cases, there was good agreement between the wild-type *K*_D_ (or ΔG°) values when obtained through individual or global fits as the average difference between individual and global fitting for wild-type measurements was 0.1 kcal/mol. In contrast, there was substantial variation for the lower affinity mutant datasets: the average difference in ΔG°-values here was 0.8 kcal/mol.

**Figure 5:**
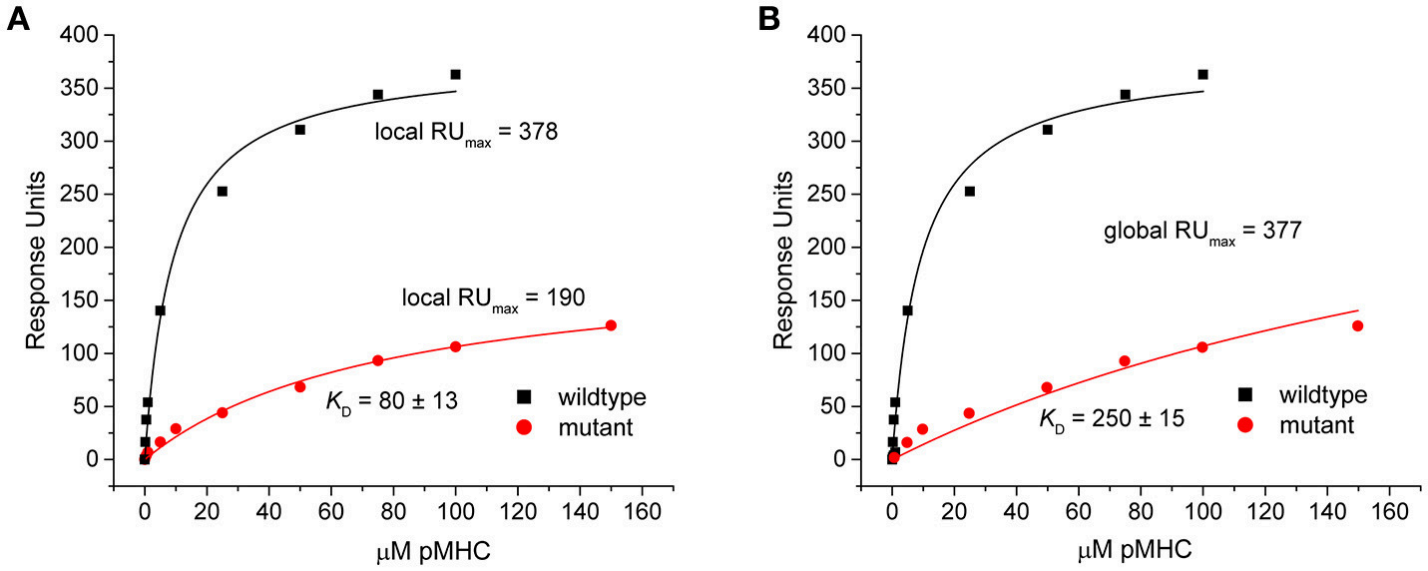
**Individual analysis of mutant datasets tends to overestimate degree of saturation, inflating ***K***_**D**_ and reducing ΔΔG°. (A)** Individual fits of the DMF5 TCR binding to wild-type (black) and mutant (red) pMHC. Although the exact same surface was used for both titrations, the fitted RU_max_ differed between the wild-type and mutant experiments. The lower value for the mutant interaction led to an inflated *K*_D_ and smaller ΔΔG°. **(B)** When both datasets are fit globally with RU_max_ as a shared parameter, the reported value is the same as the high affinity value in the wild-type single analysis. The mutant *K*_D_ was thus weaker and ΔΔG° smaller.

This discrepancy arose because when singly fit, the mutant datasets appeared to overestimate the saturation levels. This was indicated by the fitted RU_max_-values, which were reduced by nearly half in the eight outlier data sets in Figure 3 (RU_max_ for individually fit mutant data was on average 59% of the RU_max_-value in the wild-type or globally fit data). As lower-than-true RU_max_-values indicated greater saturation, this led to stronger mutant *K*_D_-values. An example of this occurrence is shown in Figure 5 for dataset 17 of the experimental data. Dataset 17 involves a variant of the DMF5 TCR interacting with both wild-type and mutant HLA-A2 protein presenting the MART1 peptide. In the titration with wild-type HLA-A2, good saturation is achieved, yielding a RU_max_ of 378 ± 14. However, when mutant HLA-A2 complex is injected over the same surface, the individual fit to the data yields a reduced RU_max_ of 190 ± 15. The apparent increase in saturation yields a *K*_D_ of 80 ± 13 μM, for a ΔΔG° of −1.27 ± 0.15 kcal. With global fitting, the RU_max_ for the mutant is determined at 377 ± 11. The *K*_*D*_ for the mutant interaction is then determined instead to be 250 ± 15 μM, and the ΔΔG° is −1.97 ± 0.09 kcal/mol.

This tendency to overestimate the degree of saturation and thus affinities of mutant interactions was not observed with the individually fit, error-modified simulated data, despite varying the ΔΔG° between high and low affinity data and adding additional noise. While individually fit simulated data did show greater fitting errors and larger ranges for the recovered mutant ΔG°-values (off by 2- to 5-fold from the actual value value), there was an even distribution between values above and below the actual ΔG°-value. The fitted RU_max_-values matched this trend: approximately half of the noisy data sets had RU_max_-values higher than the actual value while half were lower.

The reason for the apparent enhancement in saturation for the lower affinity experimental data could have multiple causes. As all data were collected using SPR, it is possible that the sensor chip surface is deteriorating during the experiment. However, this would be expected to impact both high and low affinity experiments, and should impact both global and individual fits, which was not observed. Further, control experiments altering the order of injections (i.e., mutant then wild-type) and TCR vs. pMHC orientations on the sensor surface did not alter the outcome. A more likely possibility is that injection of high protein concentrations leads to surface aggregation, blocking available receptor binding sites and altering the shape of the curve, while simultaneously leading to higher RU units with each injection. As the curve shape and saturation point is restricted by the greater information content (curvature) in the higher affinity data, global fitting would be less subject to this occurrence. This is related to the “pseudo-saturation” profiles discussed by Myszka and colleagues in their review of SPR literature (Rich and Myszka, 2006, 2008). Regardless of the underlying causes, however, the results are clear that global fitting minimizes the impact of experimental artifacts present in SPR-based analysis of wild-type/mutant TCR–pMHC interactions.

### Extending the applicability of global analysis to perform double mutant cycle experiments

Double mutant cycle experiments are frequently used to determine the interaction free energy between two sidechains (Horovitz, 1996). Double mutant cycles can be particularly helpful in studying TCR–pMHC interactions, as they allow investigators to isolate side-chain interactions and study interactions between separate components of the interface, such as the peptide, MHC α helices, or the various TCR CDR loops (Piepenbrink et al., 2013; Blevins et al., 2016). Double mutant cycles require three ΔΔG° measurements: two with two single mutants (one on the TCR, one on the pMHC), and one with a double mutant (mutations on both molecules). As a construct of ΔΔG° measurements emerging from four separate binding experiments, global analysis can therefore be used to enhance accuracy and precision of double mutant cycles.

**Figure 6:**
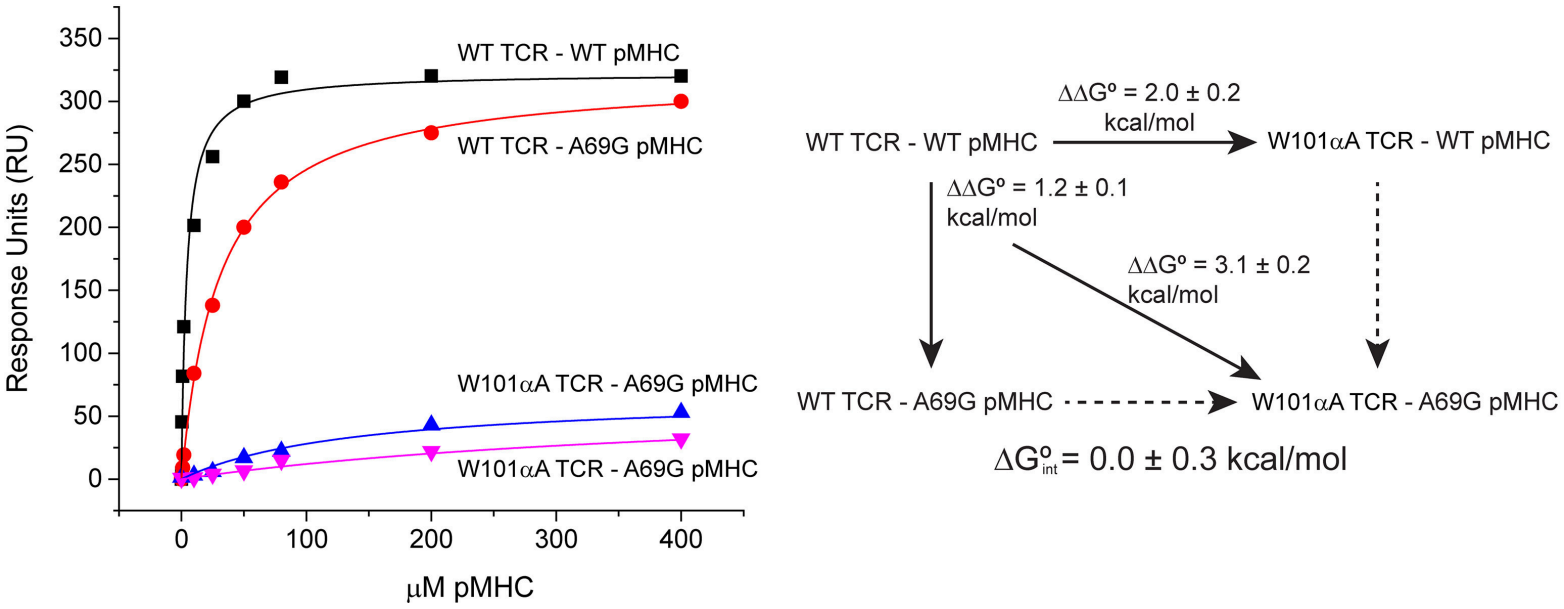
**Example of a double mutant cycle analysis in a TCR-pMHC interface**. When the double mutant cycle exploring the interaction between Trp101 of the A6 TCR and Ala69 of HLA-A2 was fit globally, the reported interaction free energy was 0.0 ± 0.3 kcal/mol. If the four datasets making up the interaction were individually fit, the resulting interaction free energy was −1.0 ± 0.2 kcal/mol.

Global analysis of SPR-based double mutant cycle experiments is simply an extension of the ΔΔG° measurements described above. Both wild-type and mutant TCRs can be coupled to adjacent flow cells, over which wild-type and mutant pMHC will be injected. This design allows for parameter sharing, with the RU_max_ constrained for both surfaces. A single global fit can thus be used for all four ΔG° measurements that make up the cycle. An example is shown in Figure 6, which illustrates a double mutant cycle measuring the interaction between Trp101of CDR3α of the A6 TCR and Ala69 of the α1 helix of HLA-A2 presenting the Tax peptide. The four data sets are fit simultaneously, and the free energy of interaction between the two side chains is measured as 0.0 ± 0.3 kcal/mol. Fitting each experiment independently yields a very different value of −1.0 ± 0.2 kcal/mol, but is subject to the errors and complexities described above.

Previously published data further illustrates the robustness of global analysis in double mutant cycles (Piepenbrink et al., 2013). The free energy of interaction between Glu30 of CDR1α of the A6 TCR and Tyr8 of the Tax peptide presented by HLA-A2 was measured as −1.7 ± 0.1 kcal/mol. In this case the ΔΔG° for the double mutant interaction was very weak. This measurement was therefore repeated in the background of a variant of the A6 TCR that bound with 100-fold higher affinity (Haidar et al., 2009; Cole et al., 2013). The resulting free energy of interaction was −1.6 ± 0.2 kcal/mol, identical within error to the first measurement.

### Concluding remarks

Here, we demonstrated how global analysis of multiple binding datasets can be used to substantially extend the accuracy and precision of TCR-pMHC binding experiments. We emphasized SPR experiments, which continue to dominate quantitative assessments of TCR-pMHC binding. However, the approaches described herein can be applied to other experimental techniques, such as calorimetric and fluorescence experiments (Piepenbrink et al., 2009b). Beyond improving ΔΔG° measurements, the approach facilitates more complex experimental designs such as double mutant cycles, which provide the opportunity for novel insight into TCR recognition properties. As experimental studies of TCR-pMHC binding segues into rational design for new biologics and therapeutics, which often require the generation of large training sets (Riley et al., 2016), the improved reliability available through global analysis should be expected to have a direct influence on progress.

## Materials and methods

### Creation and perturbation of simulated data

To determine the accuracy of ΔΔG°-values obtained by either single or global fitting methods, a simulated data set was created. An RU_max_ of 150 was selected along with *K*_D_-values of 2 and 100 μM for the wild-type and mutant interaction, respectively. Ten pMHC concentration points were selected ranging from 0.25 to 100 μM and response units calculated for the two TCR-pMHC interactions using a simple 1:1 binding model.

Simulated data was subjected to Gaussian-distributed noise ranging from 5 to 30% of the response values in 2.5% steps, applied to each data point. Error was added using the “white_noise” function in Origin 9.0. These noisy datasets were fit individually and globally to compare effects on ΔΔG°-value and error. Averaging the reduced χ^2^-values from 25 experimental TCR-pMHC SPR fits allowed for an estimate of the noise typically seen in experimental data (amounting to 22% of the RU_max_). To mimic this, the simulated data was perturbed by adding Gaussian-distributed noise ranging from 5 to 30% of the 150 RU_max_ as shown in Table 1.

Additional simulated datasets were created to explore effects on affinity and the spread of ΔΔG°. These sets included widening the spread between the high and low affinity interactions and using the RU_max_- and *K*_D_-values from an experimental dataset to develop a simulated set. This data was also subjected to both single and global fitting as well as noise and error propagation.

### Fitting data using individual and global fitting methods

Datasets were fit using both individual and global fitting. For individual fitting, both RU_max_ and ΔG° were fitted parameters. For global fitting, RU_max_ was a shared parameter between the high and low affinity interactions while ΔG°-values were individually floated. For global fitting, both wild-type and mutant datasets were fit simultaneously using a custom function in Origin 9.0. Errors were calculated by standard error propagation using the fitting errors obtained from Origin. Student's *t*-tests were performed to ensure differences between individual and global fitting were statistically different. *F*-tests were used to quantify differences in distribution of ΔΔG°-values and error.

### Collection of experimental data and perturbations

Twenty-five sets of TCR-pMHC binding experiments were utilized (Table S1). Ten of the datasets were from previously published work using double mutant cycles (Piepenbrink et al., 2013; Blevins et al., 2016), whereas 15 new datasets were collected. Pairs of these datasets were used for “wild-type” and “mutant” conditions to create ΔΔG°-values. In addition to serving as primary data, these data were subjected to perturbations, including adding additional Gaussian-distributed noise and removing data points as shown in Table 1. For the eight data sets with ΔΔG° differences outside the threshold of ±0.62 kcal/mol, averages and standard deviations for the whole set of 72 perturbed data sets were obtained by calculating the average ΔΔG°-value and standard deviation for each experimental sample, and then averaging the eight resulting averages and standard deviations.

### Surface plasmon resonance to determine binding affinity

Soluble TCRs and pMHC complexes were produced from bacterially expressed inclusion bodies and purified as previously described (Davis-Harrison et al., 2005). Experiments to determine binding affinity were performed as previously described unless otherwise noted (Davis-Harrison et al., 2005). Briefly, steady state equilibrium binding experiments were performed on a Biacore 3000 instrument. Purified TCR was diluted in 10 mM sodium acetate pH 4.0 and immobilized on the surface of a CM5 chip to a density of ~1000 RU. Purified pMHC was serially injected over the surface in concentrations ranging from 0.5 to 200 μM in duplicate. Experiments were carried out at 25°C. Alternatively, pMHC was immobilized on the chip to densities of ~2000 RU and TCR was serially injected over the surface in concentrations ranging from 0.5 to 300 μM. These experiments were performed at 10°C. All data was first processed in BiaEvaluation 4.1 prior to fitting in Origin 9.0.

Double-mutant cycles were performed analyzed as described previously (Piepenbrink et al., 2013; Blevins et al., 2016). Wild-type TCR and mutant TCR were immobilized on the surface of a CM5 chip. Soluble wild-type and mutant pMHC complexes were injected over the chip surface in a series of increasing concentration points until steady-state binding was attained. Each injection series was repeated twice. In any one experiment, three flow cells were used: a blank, wild-type TCR, and mutant TCR. The output of one single experiment thus consisted of two sets of all four measurements that comprise a double-mutant cycle. Data were processed in BiaEvaluation 4.1, and all eight datasets were simultaneously analyzed in Origin 9.0 using a custom function. The wild-type and mutant TCR surface densities and the four ΔG°-values that make up the cycle were parameters in the global fitting function. Data sets that shared a common flow cell shared an RU_max_-value, with the *K*_D_- and ΔG°-values being fit locally. Errors were propagated using standard statistical error propagation methods.

## Author contributions

SB performed experiments and analyzed data. BB oversaw the project. Both authors wrote and edited the manuscript.

## Funding

Supported by grant R35GM118166 from the National Institute of General Medical Sciences, National Institutes of Health.

### Conflict of interest statement

The authors declare that the research was conducted in the absence of any commercial or financial relationships that could be construed as a potential conflict of interest. The reviewer JZ and handling Editor declared their shared affiliation, and the handling Editor states that the process nevertheless met the standards of a fair and objective review
