# Supplementary material for: Using Global Analysis to Extend the Accuracy and Precision of Binding Measurements with T cell Receptors and Their Peptide/MHC Ligands
Source: Front Mol Biosci. 2017 Jan 31;4:2. doi: 10.3389/fmolb.2017.00002 (PMC5281623; doi:10.3389/fmolb.2017.00002)
Supplement: Supplementary file 1 [file Table1.pdf]

Table S1. Pairs of experimental "wild-type" and "mutant" experiments used. Pairs were collected with the same surface to facilitate sharing of RU_{max}.

Dataset	"Wild-type" (higher affinity) interaction		"Mutant" (lower affinity) interaction		ΔΔG° individual	ΔΔG° global	abs[Δ(ΔΔG°)]	Ref [c]
	TCR [a]	pMHC [b]	TCR [b]	pMHC [a]				
1	A6	Tax/HLA	A6	Tax/HLA A69G	-1.17	-1.18	0.01	*1*
2	A6 αW101A	Tax/HLA	A6 αW101A	Tax/HLA A69G	0.05	-0.68	0.73	*1*
3	DMF5 βL98W	ELA/HLA	DMF5 βL98W	ELA/HLA Q155A	-0.95	-0.98	0.04	
4	DMF5 αD26Y βL98W	ELA/HLA	DMF5 αD26Y βL98W	ELA/HLA Q155A	-0.55	-0.76	0.21	
5	DMF5 αD26Y βL98W	ELA/HLA	DMF5 αD26Y βL98W	ELA/HLA Q155A	-0.71	-1.44	0.72	
6	DMF5 αD26Y/Y50A βL98W	ELA/HLA	DMF5 αD26Y/Y50A βL98W	ELA/HLA Q155A	-0.53	-0.79	0.26	
7	DMF5	ELA/HLA	DMF5	ELA/HLA Q155A	-0.81	-1.25	0.45	*1*
8	DMF5 αD26Y/Y50A βL98W	ELA/HLA	DMF5 αD26Y/Y50A βL98W	ELA/HLA Q155A	-0.31	-0.65	0.34	*1*
9	DMF5	ELA/HLA	DMF5	ELA/HLA E166A	-0.99	-1.22	0.23	*1*
10	DMF5 αD26Y/N52A βL98W	ELA/HLA	DMF5 αD26Y/N52A βL98W	ELA/HLA E166A	-0.73	-1.55	0.83	*1*
11	Mel5 β3	FAT/HLA	Mel5 β3	FAT/HLA Q155A	-1.97	-1.66	0.31	
12	Mel5 αY50A β3	FAT/HLA	Mel5 αY50A β3	FAT/HLA Q155A	-0.42	-0.32	0.1	
13	A6	Tax/HLA	A6	$Y5F^{3,4ff}$ Tax/HLA	0.19	0.55	0.35	
14	A6	Tax/HLA	A6	$Y5F^{3,4ff}$ Tax/HLA	0.48	0.54	0.05	
15	A6 βH29A	Tax/HLA	A6 βH29A	$Y5F^{3,4ff}$ Tax/HLA	0.13	0.71	0.58	
16	A6 βH29A	Tax/HLA	A6 βH29A	$Y5F^{3,4ff}$ Tax/HLA	0.25	0.67	0.42	
17	DMF5 αD26Y βL98W	ELA/HLA	DMF5 αD26Y βL98W	ELA/HLA R65A	-1.27	-1.97	0.69	*2*
18	DMF5 αD26Y βY47A/L98W	ELA/HLA	DMF5 αD26Y βY47A/L98W	ELA/HLA R65A	-1.15	-1.58	0.43	*2*
19	DMF5 αD26A βL98W	ELA/HLA	DMF5 αD26A βL98W	ELA/HLA R65A	-1.08	-3.01	1.94	*2*
20	DMF5 βL98W	ELA/HLA	DMF5 βL98W	ELA/HLA R65A	-1.37	-2.07	0.69	*2*
21	DMF5 αD26A βL98W	ELA/HLA	DMF5 αD26A βL98W	ELA/HLA R65A	-0.98	-1.59	0.62	
22	DMF5 αD26A βL98W	ELA/HLA	DMF5 αD26A βL98W	ELA/HLA R65A	-0.89	-1.66	0.77	
23	DMF5 αD26Y βY47A/L98W	ELA/HLA	DMF5 αD26Y βY47A/L98W	ELA/HLA R65A	-1.1	-1.6	0.5	
24	DMF5 αD26Y βY47A/L98W	ELA/HLA	DMF5 αD26Y βY47A/L98W	ELA/HLA R65A	-1.07	-1.52	0.44	
25	DMF5 αD26Y βL98W	ELA/HLA	DMF5 αD26Y βL98W	ELA/HLA R65A	-1.14	-1.43	0.23	

[a] TCR and mutations indicated. Mel5 β3 refers to a high affinity variant of the Mel5 TCR (Madura 2013).
[b] pMHC indicated along with mutation/peptide substitution. Tax refers to the Tax11-19 peptide (sequence LLFGYPVYV); ELA refers to the anchor modified MART1 decamer (sequence ELAGIGILTV). $Y5F^{3,4ff}$ and FAT refer to variants of the Tax and MART1 peptides, respectively (Piepenbrink 2009a; Ekeruche-Makinde 2012).
[c] Reference for previously published data, or new data if left blank (*1* = Piepenbrink 2013; *2* = Blevins 2016).

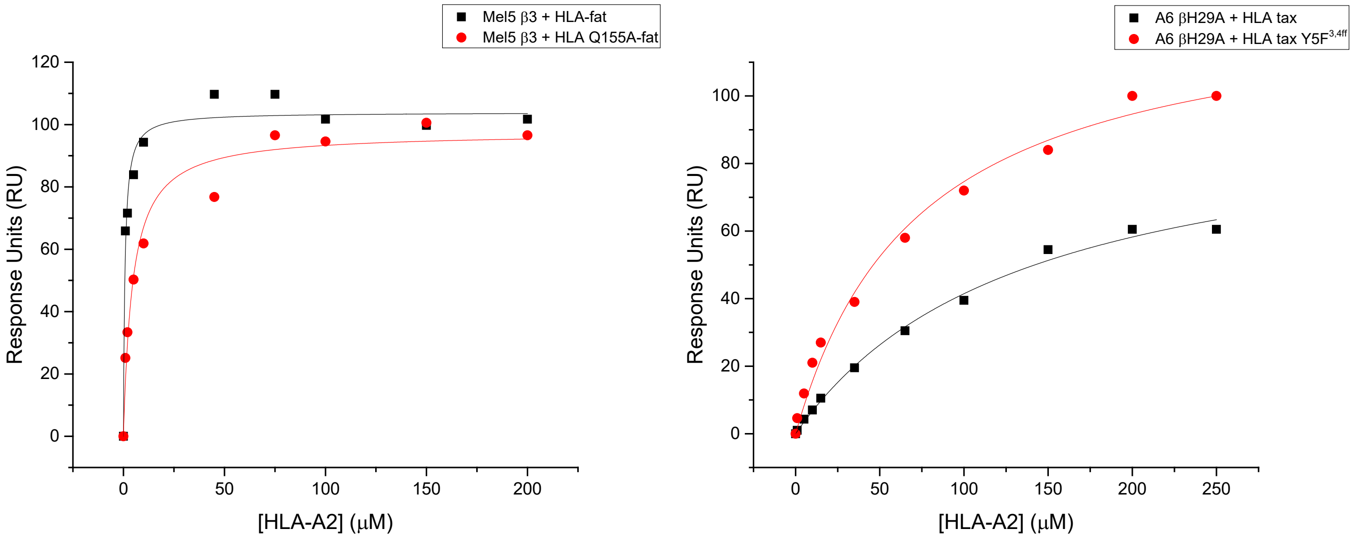

Figure S1. Representative pairs of SPR binding data for previously unpublished data in Table S1. The data on the left is for dataset 11, the data on the right is dataset 15.
